# Elevated CO_2_ impacts bell pepper growth with consequences to *Myzus persicae* life history, feeding behaviour and virus transmission ability

**DOI:** 10.1038/srep19120

**Published:** 2016-01-08

**Authors:** Beatriz Dáder, Alberto Fereres, Aránzazu Moreno, Piotr Trębicki

**Affiliations:** 1 Institute of Agricultural Sciences. Spanish National Research Council. Calle Serrano 115 dpdo, 28006 Madrid, Spain; 2 Grains Innovation Park. Department of Economic Development, Jobs, Transport and Resources. 110 Natimuk Road, Horsham VIC 3400, Australia

## Abstract

Increasing atmospheric carbon dioxide (CO_2_) impacts plant growth and metabolism. Indirectly, the performance and feeding of insects is affected by plant nutritional quality and resistance traits. Life history and feeding behaviour of *Myzus persicae* were studied on pepper plants under ambient (aCO_2_, 400 ppm) or elevated CO_2_ (eCO_2_, 650 ppm), as well as the direct impact on plant growth and leaf chemistry. Plant parameters were significantly altered by eCO_2_ with a negative impact on aphid’s life history. Their pre-reproductive period was 11% longer and fecundity decreased by 37%. Peppers fixed significantly less nitrogen, which explains the poor aphid performance. Plants were taller and had higher biomass and canopy temperature. There was decreased aphid salivation into sieve elements, but no differences in phloem ingestion, indicating that the diminished fitness could be due to poorer tissue quality and unfavourable C:N balance, and that eCO_2_ was not a factor impeding feeding. Aphid ability to transmit *Cucumber mosaic virus* (CMV) was studied by exposing source and receptor plants to ambient (427 ppm) or elevated (612 ppm) CO_2 _before or after virus inoculation. A two-fold decrease on transmission was observed when receptor plants were exposed to eCO_2_ before aphid inoculation when compared to aCO_2_.

Ambient carbon dioxide (aCO_2_) concentration has exceeded 400 ppm and future estimations predict an increase up to 550 ppm within a few decades^1^,^2^. Apart from the consequences that increasing CO_2_ is the main driver behind climate change, it plays a mayor role in plant growth, physiology and metabolism as it is the direct substrate for photosynthesis^3^. Among the observed effects on plants, typical responses to elevated CO_2_ (eCO_2_) include increased plant growth and biomass, canopy size, reduction in stomatal conductance and transpiration, improved water-use efficiency and higher photosynthetic rates^4^,^5^,^6^,^7^,^8^,^9^,^10^,^11^,^12^,^13^. At the same time, increasing CO_2_ alters the chemical composition of plant tissue, with the accumulation of non-structural carbohydrates such as soluble sugars and starch^14^,^15^,^16^. Elevated CO_2_ also has an impact on the nitrogen cycle that translates into a decrease in protein content and higher C:N ratio^7^,^15^,^17^,^18^. The reduction in stomatal conductance may lead to a decrease in micronutrients such as calcium, magnesium or phosphorus due to the lower water uptake from the soil^17^.

Indirectly, the performance and feeding behaviour of herbivorous insects is greatly affected by plant nutritional quality and resistance, which are likely to be altered by increasing CO_2_^6^,^10^,^11^,^15^,^19^. Also, changes in plant chemistry and physiology alter natural enemies^9^,^20^,^21^,^22^,^23^, as well as the incidence of plant viruses^24^,^25^,^26^,^27^. Conversely to plant growth, there is no general agreement on the effects of elevated CO_2_ on aphid-plant interactions as aphids are among the sap-feeding insects that have responded either positively or negatively to CO_2_-induced changes in plants^5^,^6^,^28^. Aphid abundance under eCO_2_ was higher in *Aphis gossypii* Glover, *Sitobion avenae* Fabricius or *Acyrthosiphon pisum* Harris on barrel medic^29^,^30^,^31^,^32^. Increasing CO_2_ was also beneficial for *Rhopalosiphum padi* L. growth rates and weight on wheat^11^,^15^,^33^.

On the contrary, a negative response was found in *A. pisum* on broad bean and *R. padi* on tall fescue^6^,^18^. *Brevicoryne brassicae* L. reduced Brussels sprout colonization after long-term exposure to eCO_2_^13^. Other effects on life history parameters included an increased proportion of *Rhopalosiphum maidis* Fitch alates^32^. Lastly, several aphid species were reported to show a neutral response, such as *Macrosiphum euphorbiae* Thomas and *Aulacorthum solani* Kaltenbach^6^,^34^,^35^. In addition, responses seem to be host- and even genotype-specific, e.g. *A. pisum* on lucerne and broad bean^6^,^35^,^36^,^37^.

*Myzus persicae* Sulzer, the aphid species studied here, is a cosmopolitan, polyphagous pest of greenhouses and field crops. More importantly, it is a highly efficient vector of more than 100 plant viruses; therefore consecutive insecticide applications to lower vector density have constituted the traditional control strategy in the past, causing environmental and energetic costs^38^. Recent findings suggest that eCO_2_ is detrimental for its progeny, growth rates and adult weight on Brassicaceae^10^,^15^,^21^,^33^, although opposite results have been found on *Solanum dulcamara* L. and *Arabidopsis thaliana* L.^6^,^39^.

Feeding behaviour of insect pests can be monitored by the Electrical Penetration Graph (EPG) technique, which provides a live visualization and recording of plant penetration by insect mouthparts^40^,^41^. Few studies have considered the feeding behaviour of aphids under rising CO_2_, but decreased aphid salivation into sieve elements, increased phloem sap ingestion and shorter non-pathway phase are among the effects observed for *A. pisum* on barrel medic^31^,^42^.

There is limited information about the consequences of increasing CO_2_ on viral dynamics. Although it has been proven that high doses provoke resistance against infection with *Tobacco mosaic virus* (TMV, *Tobamovirus*) in tomato plants^43^, and *Potato virus Y* (PVY, *Potyvirus*)^44^ and *Cucumber mosaic virus* (CMV, *Cucumovirus*) in tobacco, due to the fact that plant defences appear to deviate from viruses to aphids under increasing CO_2_^45^. On the other hand, *Barley yellow dwarf virus* (BYDV, *Luteovirus*) incidence has been predicted to increase in wheat under elevated CO_2_^27^. This would imply that the larger amount of biomass could constitute a reservoir of infected material with subsequent higher risk of virus transmission by insect vectors^24^. Whilst, symptomatology of vegetable viruses can also be affected by rising CO_2_, enhancing earlier or more pronounced phenotypic differences between healthy and infected plants and making infected hosts more attractive to vectors^26^,^27^.

Investigating plant-insect interactions under increasing CO_2_ is crucial to evaluate their consequences on environment and ecosystems, and to develop new crop management strategies under future climate change scenarios. Therefore, the objective of our work was to study the life history parameters and feeding behaviour of *M. persicae* on pepper plants under aCO_2_ (400 ppm) and eCO_2_ (650 ppm). We also analysed the direct impact of increasing CO_2_ on plant growth and leaf chemistry. Links between plant tissue quality and aphid responses are discussed. Lastly, the cuticula-borne, non-persistent virus CMV was included in this investigation. We studied CMV transmission by *M. persicae* on ambient (427 ppm) and elevated CO_2_ (612 ppm) conditions with source and receptor plants exposed to the two CO_2_ regimes. Receptor plants were exposed to CO_2_ conditions either immediately after aphid introduction (direct exposure), or two weeks prior, in order to study previous acclimation (indirect exposure).

## Results

### Aphid life history

**Figure 1:**
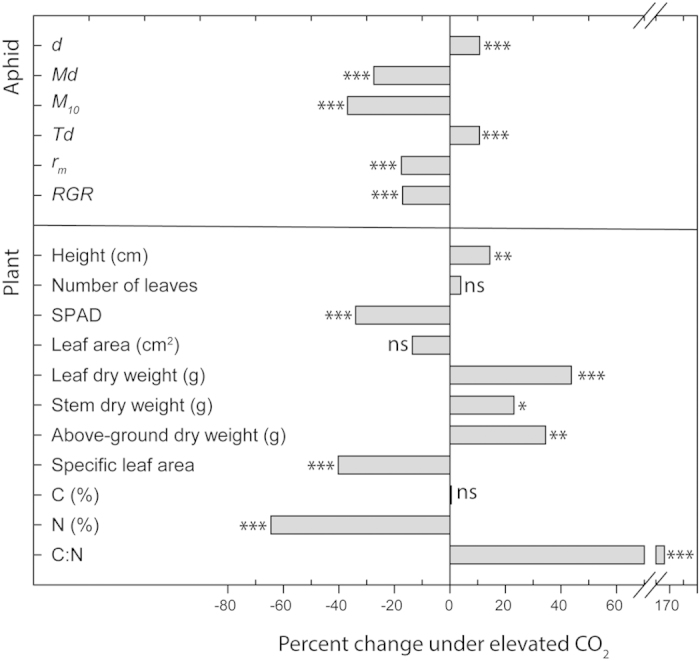
Percent change under eCO_2_ of the life history parameters of aphid (*Myzus persicae*), plant growth and leaf chemical profile. Statistical differences are calculated according to Student *t*-test for Gaussian variables or Mann-Whitney *U*-test for non-Gaussian variables (*p* ≤ 0.05). No significant differences are labelled as “ns”, and significant differences are indicated by stars with ***p ≤ 0.001, **p < 0.01 and *p < 0.05. *d* is the time (days) from birth to the onset of reproduction; *Md* is the reproductive output per aphid that represents the duration of d; *M*_*10*_ is the mean offspring number per female over the 10 day period; *Td* is the mean generation time; *r*_*m*_ is the intrinsic rate of natural increase and *RGR* is the mean relative growth rate.

**Figure 2:**
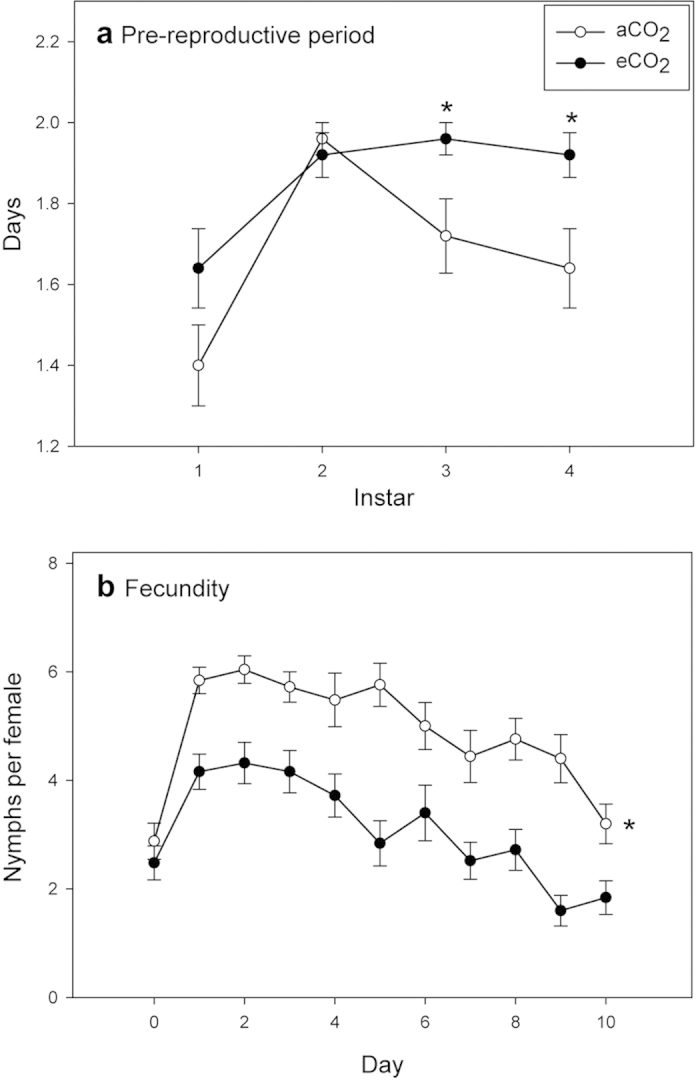
(**a**) Mean duration ± SEM of the four *Myzus persicae* nymphal instars under aCO_2_ and eCO_2_. (**b**) Mean number of daily nymphs per female ± SEM under aCO_2_ (400 ppm) and eCO_2_ (650 ppm) for 10 days. Asterisks indicate statistical differences according to (**a**) Mann-Whitney *U*-test and (**b**) GLM repeated measures analysis (*p* ≤ 0.05).

To asses the effect of increased CO_2_ concentration on pepper plants and *M. persicae*, plants were grown under aCO_2_ (400 ppm) or eCO_2_ (650 ppm). *Myzus persicae* pre-reproductive period (*d*), which was measured from aphid birth to adulthood was 11% longer on pepper plants from eCO_2_ environment (*U* = 138.5, *Z* = −3.706, *p* < 0.001) (Fig. 1, Supplementary Table S1). Additionally, under eCO_2_, instars N3 and N4 lasted significantly longer than under ambient conditions (N3: *U* = 237.5, *Z* = −2.291, *p* = 0.022. N4: *U* = 225.0, *Z* = −2.366, *p* = 0.018) (Fig. 2a). At the same time, the effective fecundity (*Md*) (*t* = 5.537, df = 48, *p* < 0.001) and offspring production over 10 days (*M*_*10*_) (*t* = 8.352, df = 48, *p* < 0.001) dropped respectively by 27% and 37% on plants grown under eCO_2_ (Fig. 1, Supplementary Table S1). Daily offspring production was also significantly lower under eCO_2_ when compared to ambient conditions (Treatment: *F* = 69.749, df = 1, *p* < 0.001. Time: *F* = 13.730, df = 10, *p* < 0.001. Treatment × Time: *F* = 1.749, df = 1, *p* = 0.067) (Fig. 2b). This latter treatment resulted in a higher mean generation time (*Td*) (*U* = 138.5, *Z* = −3.706, *p* < 0.001), as well as a slower intrinsic rate of natural increase (*r*_*m*_) (*t* = 7.407, df = 48, *p* < 0.001) and mean relative growth rate (*RGR*) (*t* = 7.407, df = 48, *p* < 0.001) when compared to aphids reared on aCO_2_ peppers (Fig. 1, Supplementary Table S1).

### Feeding behaviour by Electrical Penetration Graphs

The sequential and non-sequential variable values obtained for the stylet penetration activities of *M. persicae* on aCO_2_ and eCO_2_-grown pepper plants showed relevant differences between treatments. Aphids made significantly fewer probes (*U* = 603.500, *Z* = −2.196, *p* = 0.028), intercellular stylet pathways C (*U* = 584.000, *Z* = −2.377, *p* = 0.017), phloem salivations E1 (*U* = 582.500, *Z* = −2.415, *p* = 0.016), single E1 not followed by phloem ingestion E2 (*U* = 567.000, *Z* = −2.612, *p* = 0.009) and probes after the first E1 (*U* = 546.000, *Z* = −2.831, *p* = 0.005) on eCO_2_ peppers (Table 1). Furthermore, the total duration of E1 per insect (*U* = 598.000, *Z* = −2.089, *p* = 0.037), and the time elapsed from the end of the last short intracellular puncture pd to the end of the probe (*U* = 540.000, *Z* = −2.783, *p* = 0.005) were significantly shorter on eCO_2_ peppers (Table 1). With regard to waveform duration per event, significant differences were found for non-probe activity (*U* = 104194.500, *Z* = −3.245, *p* = 0.001) and short intracellular punctures pd (*U* = 2193221.000, *Z* = −15.843, *p* < 0.001), with aphids spending less time on these activities if reared on eCO_2_ peppers (Table 1). When the percentage of time spent on each activity was evaluated, aphids phloem salivations E1 was reduced on eCO_2_ peppers (*U* = 591.000, *Z* = −2.310, *p* = 0.021) (Table 1). No differences were found for phloem ingestion (E2) parameters.

### Plant growth and physiology

**Figure 3:**
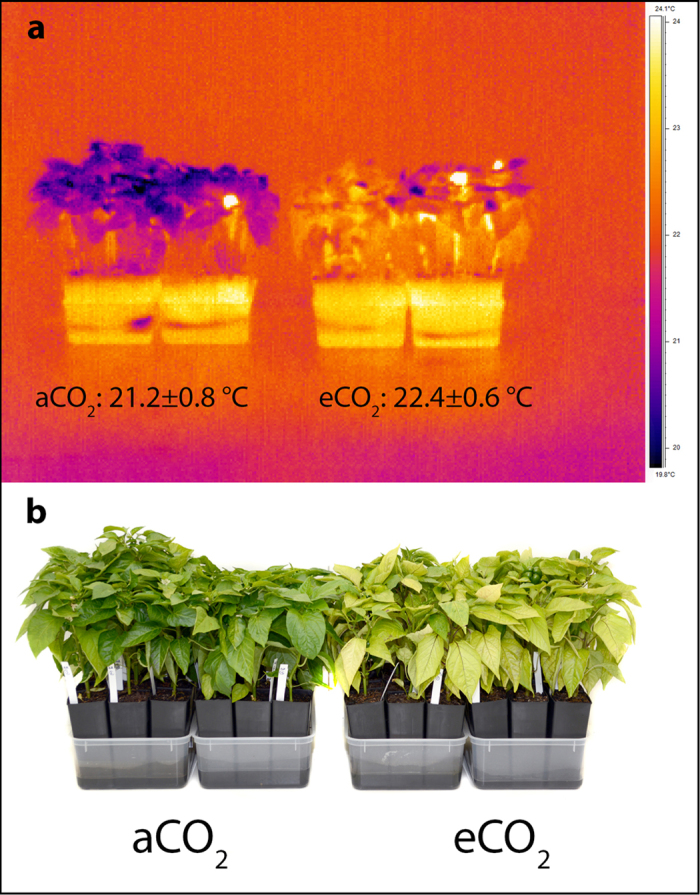
(**a**) Infrared thermal image of the canopy temperature of peppers, with mean ± SEM values measured under aCO_2_ and eCO_2_. (**b**) Picture of two-month old peppers under aCO_2_ and eCO_2_.

Peppers grown under eCO_2_ for four weeks were significantly taller (*t* = −3.277, df = 48, *p* = 0.002) although they had the same number of leaves than those under aCO_2_ (Fig. 1, Supplementary Table S1). At harvest, leaf (*t* = −3.732, df = 48, *p* = 0.001), stem (*t* = −2.454, df = 48, *p* = 0.018) and above-ground dry biomass (*t* = −3.367, df = 48, *p* = 0.002) were significantly higher under eCO_2_ (Fig. 1, Supplementary Table S1). Leaf area was similar in both treatments but there was a significant decrease in specific leaf area under eCO_2_ (*t* = 13.158, df = 48, *p* < 0.001) (Fig. 1, Supplementary Table S1). Moreover, SPAD was significantly lower under eCO_2_ (*t* = 10.044, df = 48, *p* < 0.001) (Fig. 1, Supplementary Table S1). Exposure to eCO_2_ did not alter carbon content, however it significantly decreased foliar nitrogen content (*U* = 0.000, *Z* = −5.127, *p* < 0.001) (Fig. 1, Supplementary Table S1). Additionally, the pepper canopy temperature measured using an IR camera was 1.2 °C higher under eCO_2_ (aCO_2_: 21.2 ± 0.8 °C, eCO_2_: 22.4 ± 0.6 °C) (Fig. 3a).

### CMV transmission by aphids

**Figure 4:**
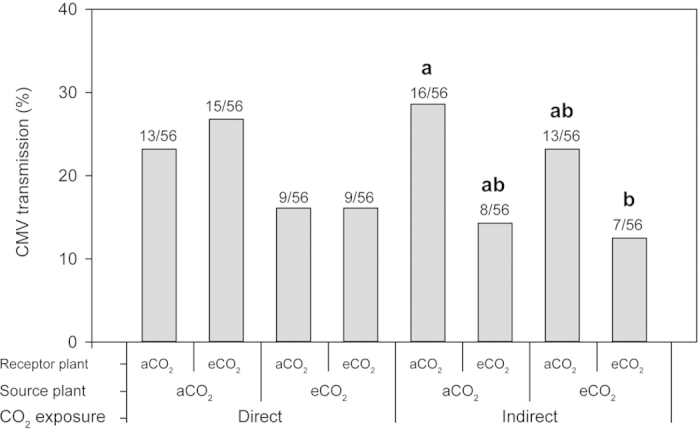
CMV transmission (%) of direct and indirect CO_2_ exposure experiments, with receptor plants exposed to the two CO_2_ regimes after and before aphid introduction, respectively. Ratios on bars refer to the number of CMV-infected receptor plants out of the total tested. Different letters in bold stand for statistical differences according to a χ^2^ goodness of fit test (*p* ≤ 0.05).

To understand the potential effects of increased CO_2_ on virus transmission/acquisition efficiency, we used CMV and the same aphid/plant combination as per previous experiments. No differences were found if CO_2_ was applied to receptor plants straight after aphid introduction (direct exposure), when the CMV source inoculum had been grown either under aCO_2_ or eCO_2_ (Fig. 4). If the receptor plants had been previously grown under one of the CO_2_ regimes before CMV inoculation by aphids (indirect exposure), a two-fold decrease on CMV transmission was observed when source and receptor plants were grown under eCO_2_ conditions compared to aCO_2_ (χ^2^ = 4.432, *p* = 0.035) (Fig. 4).

## Discussion

Current carbon dioxide (CO_2_) concentration has exceeded 400 ppm and the concentration will continue increasing at an accelerating rate from decade to decade. This rising of CO_2_ levels in the Earth’s atmosphere causes general climate change and global warming^46^. Among the observed effects on plants, increasing CO_2_ leads to changes in plant growth, physiology and metabolism^7^,^13^,^15^. At the same time, these changes have an indirect impact on insect pest biology and behaviour, altering their population growth or feeding habits, as well as on the symptomatology and incidence of associated pathogens such as plant viruses^24^,^26^,^27^,^35^. To our knowledge, this is the first study to demonstrate the variety of effects of increasing CO_2_ on pepper plants and the subsequent indirect response on *M. persicae* fitness and feeding behavior. Moreover, these experiments have been coupled with research on CMV transmission with receptor plants exposed to ambient and elevated CO_2_ either before or after aphid introduction.

Overall *M. persicae* fitness was reduced on pepper plants grown under eCO_2_, being consistent with previous results for this aphid on Brassicaceae species^10^,^15^,^21^,^33^ and other aphid species such as *A. pisum* on broad bean or *B. brassicae* on Brussels sprout^6^,^13^,^18^. Not only was the time from birth to adulthood longer, but they also produced less offspring, which resulted in lowered growth rates. Again, this highlights the specificity of responses to hosts, as eCO_2_ has been reported to increase the abundance of the green peach aphid on herbaceous species in the past^6^,^39^. The response to eCO_2_ also differs among insect guilds. It is generally agreed that the decline in the foliar nitrogen content of host plants under eCO_2_ prolongs the development of chewing insects, and that they compensate for decreased nutritional quality by consuming more foliage^5^,^22^,^47^. By contrast, eCO_2_ has species-specific effects on phloem-sucking insects^22^.

The amount of foliar nitrogen in pepper was significantly lower under eCO_2_ when compare to ambient conditions, which may well explain the poor aphid performance we observed. Aphids which fed on plants grown under eCO_2_ had a reduced efficiency, with an 11% longer pre-reproductive period and 37% fewer nymphs. This is probably mediated by reduced nitrogen content in the pepper source as aphids tend to be limited by the amount of nitrogen rather than carbohydrates^47^. Additionally, it has been shown that this aphid species is particularly sensitive to low nitrogen contents^48^,^49^. However, it has to be also pointed out that we measured whole foliar tissue nitrogen rather than phloem nitrogen but it is likely that our results could be extended to sap composition^11^,^42^,^50^. Chlorophyll content of leaves was also significantly lower under eCO_2_, this result being consistent with significantly lower nitrogen^7^,^15^,^17^,^18^. Also, it has been reported that C:N ratio of leaves can be more affected by increasing CO_2_ than that of the plant as a whole^51^.

Carbon levels were similar in both treatments, as opposed to other plant species under eCO_2_, in which carbon content increased^14^,^15^,^16^. As expected, the increased height and above-ground dry weight of peppers are in agreement with previous studies, reporting enhanced growth under eCO_2_ in several plant species^3^,^11^,^12^,^13^,^21^,^33^,^35^,^36^. Finally, using infrared imagery an increase of 1.2 °C in plant canopy temperature was measured for peppers grown under eCO_2_. Cotton and wheat also show an increase ranging from 0.6 to 1.1 °C, and closure of leaf stomata appears to be the main driver behind this response^52^. Increased plant canopy temperature can further modify the microclimate for the aphid and higher temperature has been shown to increase virus titer in wheat^53^.

In this study, EPG recordings showed decreased aphid salivation into sieve elements and shorter non-probe phase under rising CO_2_, similar to the effects found for *A. pisum*^31^,^42^. However, no differences were found for the duration and number of events of phloem ingestion, suggesting that eCO_2_ was not a factor impeding sap feeding by *M. persicae*, as opposed to *A. pisum*^31^. This result may also indicate that the diminished fitness observed could be mostly due to poorer tissue quality. What analysed parameters seem to stress is that there were no differences in feeding behaviour explaining a worse performance in eCO_2_ plants. That is, aphids did not reduce or increase feeding, but simply ingested sap that was less nutritious. Therefore, with equal ingested sap, aphids grew slower under eCO_2_ because they acquired a much more unfavourable N content diet.

Furthermore, the number of single E1 waveforms (phloem salivation) not followed by E2 events (phloem ingestion) and the percentage of time spent on E1 were lower under eCO_2_. Altogether, these results might imply that the ingestion success rate once aphids reached the phloem was higher under eCO_2_. An explanation could be found in the natural defense responses triggered under herbivory pressure, resulting in a rejection of sustained feeding under ambient conditions. Indeed, eCO_2_ is likely to reduce the resistance ability of hosts against aphids by the down-regulation of genes involved in plant defence^31^,^39^,^54^. Without these barriers, aphids might be able to ingest phloem more easily under eCO_2_. Several mechanisms have been proposed, and include but are not limited to: 1) decreased activity of enzymes mediating the accumulation of reactive oxygen species, 2) reduced polymerization of phenolics absorbed by aphids that causes browning of cells in contact with the saliva, a disadvantage for aphid feeding, 3) down-regulation of ethylene signalling pathways^31^, and 4) decreased jasmonic and salicylic acid defences^39^,^54^.

Plants used for EPG recordings were grown inside CO_2_ chambers for four weeks, whereas those used for the life history experiment were exposed to CO_2_ during the aphid cycle as well, reaching a total of seven weeks, so it is likely that the nutritional profile of these plants declined with time. Additional work testing different periods of CO_2_ exposure should be addressed to evaluate if aphid response changes with time.

Work on the interactions between pepper plants and aphids was complemented with the study of CMV transmission by *M. persicae* when source and receptor plants were either previously exposed to ambient and elevated CO_2_ before aphid introduction (indirect effect) or straight after insect inoculation (direct effect). Elevated CO_2_ lessened virus transmission risk if receptor plants had been previously grown under eCO_2_, but not if CO_2_ was applied directly after pest introduction, which suggests that this difference may be associated with early exposure to elevated CO_2_. CMV is transmitted in a non-circulative manner during brief probes in the host epidermis^55^. As we observed a similar number of stylet punctures (pd) under both treatments, but shorter duration under eCO_2_, the decrease in CMV transmission might be due to plant resistance mechanisms once the virus infected the host, rather than an altered aphid feeding behaviour during the stages involved in virus inoculation.

Under ambient CO_2_ conditions, it has been shown that plant pathogens are responsible for triggering changes in nutritional quality or mediating attractiveness to increase the chances of vectors spreading viruses^56^. In our experiments, the decreased CMV transmission found under eCO_2_ may be associated with lower aphid attraction to virus-infected plants, resulting in fewer viruliferous aphids, which would ultimately reduce the spread of CMV under future climate conditions.

Consecutive rising of CO_2_ concentration and global temperature are a reality of future scenarios. If disease severity changes under forthcoming environmental conditions, then additional research becomes imperative to understand the impact of viral infections on crop production and to minimise losses due to vector infestation. Additional studies could add to knowledge on insect-plant interactions under elevated CO_2_. Further studies with other insect vectors and beneficials are required to identify if the causal factors for the observed changes are conserved among different insect guilds. With regard to subsequent virus transmission by vectors, studying the causes of a delay in symptom expression after CMV infection and the decrease in CMV transmission under indirect eCO_2_ conditions may benefit the overall knowledge on the interactions among pathogens, host plants and vectors and help predict potential outbreaks.

## Methods

### Plants and CO_2_ growth chambers

Pepper seeds (*Capsicum annum* L.) cv. ‘California Wonder’ (D. T. Brown & Co Ltd., South Windsor, Australia) were sown in plastic pots filled with potting mix containing slow release fertilizer, additional trace elements, iron and lime. At two true-leaf stage, pots were placed inside growth chambers (internal dimensions 1.2 × 0.7 × 1.5 m, 1260 L) with five 400W high pressure sodium lights (Lucagrow GE Lighting, Richmond, Australia) and four 77W halogen lights (Osram Pty. Ltd., Sydney, Australia) at two different CO_2_ regimes, aCO_2_ (400 ppm) and eCO_2_ (650 ppm) (CLIMATRON-1260, Thermoline Scientific, Smithfield, Australia). Growing conditions were 24 °C, 70% RH, 16-hour photoperiod and 1000 μmol m^−2^ s^−1^ light intensity at canopy level. Water in trays was maintained at a similar level between treatments and plants were rotated within the chambers to ensure there was no positional influence. CO_2_ regime was switched between chambers once a week to ensure there was no potential chamber effect.

### Aphid population

A clonal source population of *M. persicae* was established from a virus free female naturally infesting marshmallow (*Malva parviflora* L.) at Grains Innovation Park (Horsham, Australia). Aphids were continuously reared on pepper plants in a chamber at 22 °C, 70% RH, 16-hour photoperiod and 800 μmol m^−2 ^s^−1^ light intensity. Individuals were synchronised prior to assays to ensure they were the same age.

### Aphid life history

Peppers were previously grown under aCO_2_ (400 ppm) and eCO_2_ (650 ppm) for four weeks since two-leaf stage. A single wingless *M. persicae* adult was placed in a clip-cage on the adaxial side of the youngest fully developed leaf of each pepper and allowed to produce nymphs for 24 hours. Surplus nymphs were then removed leaving one nymph per plant, which was monitored until adulthood. Offspring were counted by removing nymphs daily for 10 days after the onset of reproduction. Duration of the four nymphal instars, pre-reproductive period (*d*), effective fecundity (offspring for a period equal to the pre-reproductive period (*Md*)), offspring for 10 days, mean generation time (*Td* = *d*/0.738), intrinsic rate of natural increase (*r*_*m*_ = 0.738*(log_e_*Md*)/*d*) and mean relative growth rate (*RGR* = *r*_*m*_/0.86) were calculated (n = 25).

### Feeding behaviour by Electrical Penetration Graphs

*Myzus persicae* probing and feeding behaviour was monitored by EPG, a tool for determining the activities of the aphid’s stylets, including probing, salivation into sieve elements and passive uptake of phloem in real time^40^,^57^. Recordings were conducted inside a Faraday cage to avoid noise and interference for eight hours on peppers previously grown under aCO_2_ (400 ppm) and eCO_2_ (650 ppm) for four weeks from the two-leaf stage. Each recording was conducted using a new plant and aphid. Recordings were made simultaneously over an eight-week period (n = 42 for aCO_2_ and n = 40 for eCO_2_). Young adult aphids were immobilized using a vacuum device^57^. A thin gold wire (12.5 μm diameter, 3 cm length) was attached to the dorsum of the insect with a small droplet of water-based silver glue (EPG-Systems, Wageningen, The Netherlands) using an entomological pin. The gold wire was glued to a copper wire (0.2 m diameter) attached to a brass pin, which was inserted into the input connector of the first-stage amplifier. The output electrode was a copper post (10 cm × 2 mm), which was inserted into the pot. Recordings were performed using a Giga-8 DC amplifier with a 1 Giga Ω input resistance (EPG-Systems, Wageningen, The Netherlands). EPG output was set to 50x gain and data was acquired at 100 Hz using a DATAQ Di700 A/D data acquisition USB device card (Dataq Instruments, Ohio, USA). Data was analysed with Stylet + a software (EPG-Systems, Wageningen, The Netherlands). All behavioural variables were then processed using the MS Excel Workbook for automatic EPG data calculation^58^.

Selected variables were compared between aCO_2_ and eCO_2_^59^: PPW, proportion of individuals that produced a specific waveform type; NWEI, number of waveform events per insect, that is the sum of the number of events of a particular waveform divided by the total number of insects under each treatment; WDI, waveform duration per insect, that is the sum of durations of each event of a particular wave-form made by each individual insect that produced that waveform divided by the number of insects that performed that particular waveform under each treatment; and WDE, waveform duration per event, that is the sum of the duration of the events for a particular waveform divided by the total number of events of that particular waveform under each treatment.

### Pepper growth and physiology

Peppers grown under aCO_2_ and eCO_2_ were monitored for height, number of leaves and leaf chlorophyll content before insect introduction. Chlorophyll was measured in SPAD (Soil Plant Analytical Development) units, commonly used as an indirect indicator of nitrogen foliar content (Chlorophyll meter SPAD-502Plus, Konica Minolta, Japan). This device measures leaf transmittance at two wavelengths, 660 and 940 nm. SPAD readings were taken on every leaf and averaged per plant.

Canopy temperature was recorded by infrared thermal imaging (ThermaCAM P40, Flir Systems Pty. Ltd., Notting Hill, Australia) at the end of the fitness experiment. Thermal imaging was taken within five minutes after plants were removed from the chambers and placed in a climate controlled room (matching chamber temperatures) and against a contrast temperature background. Images were processed, and minimum, maximum and average temperature values were recorded for each treatment. Using box area tool, an equal sized rectangle was overlaid over each treatment, which covered around 50% of the plant canopy (ThermaCAM Researcher Professional 2.8 software, Flir Systems Pty. Ltd., Notting Hill, Australia).

Plants were harvested and leaf area recorded (Portable area meter LI-300C, LI-COR Biosciences, Nebraska, USA). Leaves and stems were dried at 65 °C for 48 hours (Dehydrating oven TD-150FTS, Thermoline Scientific, Smithfield, Australia). Specific leaf area (SLA = leaf area/leaf dry weight) was calculated. Leaves were finely ground (<0.5 mm) (Tissuelyser MM300, Quiagen Retsch, Haan, Germany). Total carbon (C) and nitrogen (N) concentration of leaf tissue was determined by the Dumas combustion method using a CHN analyser (CHN 2000, LECO, St. Joseph, USA) at the University of Melbourne, Creswick. C:N ratio was calculated by dividing the concentration of C by the concentration of N for each leaf sample.

### Aphid ability to transmit CMV and systemic infection

Pepper seeds cv. ‘California Wonder’ (Ramiro Arnedo S.A., La Rioja, Spain) were sown in plastic pots filled with a 1:1 mixture of substrate (Kekkilä Iberia, Quart de Poblet, Spain) and vermiculite (No. 3, Asfaltex S.A., Barcelona, Spain). Plants were watered three times a week using 20:20:20 (N:P:K) Nutrichem 60 fertiliser at a dose of 3 g L^−1^ (Miller Chemical & Fertilizer Corp., Pennsylvania, USA).

### CMV source plants

Seedlings were infected with CMV strain M6, subgroup IA at two-true leaf stage using a non-persistent virus type protocol. Fifteen *M. persicae* with one-hour starvation were allowed to feed for five minutes (acquisition access period) on a CMV positive leaf and were transferred to the seedlings for 24 hours (inoculation access period). These virus sources were then maintained either under aCO_2_ (427 ppm) or eCO_2_ (612 ppm) conditions for four weeks until symptom development inside climate chambers with 24:20 °C (D:N), 70% RH and 14-hour photoperiod. After four weeks, these plants were used as CMV inoculum for the transmission experiments.

### CMV transmission experiments

Two types of experiments were designed based on the moment of CO_2 _exposure of receptor plants: 1) an experiment where receptor plants were exposed to the CO_2_ regimes, either aCO_2_ or eCO_2_, straight after virus inoculation by aphids, with the objective of studying the direct effect of CO_2_ on the development of the viral disease, and b) an experiment where receptor plants had been previously grown for two weeks under each of the two CO_2_ regimes before aphid introduction, to study the effect of the indirect acclimation of receptor plants on CMV transmission. In both experiments, plants providing CMV inoculum were the virus sources obtained in section 2.6.1, grown either under aCO_2_ or eCO_2_.

CMV inoculation of receptor plants for both experiments was done with the same procedure mentioned above, placing five *M. persicae* which had previously fed on the CMV source plants for five minutes onto each receptor plant (n = 56). After 24 hours, receptor plants were sprayed with imidacloprid (Confidor® 20 LS, Bayer CropScience, Valencia, Spain) and maintained either under aCO_2_ or eCO_2_ conditions inside climate chambers with 24:20 °C (D:N), 70% RH and 14-hour photoperiod for four weeks until visual inspection of symptoms.

### Statistical analysis

Data was transformed with either √(x + 0.5), x^2^, Ln(x + 1) or 2*arcsin√x if needed to reduce heteroscedasticity. Differences in parameters of aphid life history, EPG recordings and plant physiology between CO_2_ treatments were assessed with Student *t*-test (*p* ≤ 0.05) using IBM Statistics SPSS 21.0 software (SPSS Inc.). When data did not follow the ANOVA assumptions, a non-parametric Mann-Whitney *U*-test (*p* ≤ 0.05) was performed. Daily offspring for 10 days over the fitness assay was assessed with GLM repeated measures analysis (*p* ≤ 0.05) (SPSS Inc.). CMV transmission was compared by a χ^2^ goodness of fit test (*p* ≤ 0.05) to check if the observed frequency distribution was related to the expected frequency distribution using Statview 4.01 software (Abacus Concepts Inc.).

## Additional Information

**How to cite this article**: Dáder, B. *et al.* Elevated CO_2_ impacts bell pepper growth with consequences to *Myzus persicae* life history, feeding behaviour and virus transmission ability. *Sci. Rep.* **6**, 19120; doi: 10.1038/srep19120 (2016).

## Supplementary Material

Supplementary Information

## Figures and Tables

**Table 1:** Mean ± SEM (ranges in parenthesis) values of non-sequential and sequential EPG variables for the probing behaviour of *Myzus persicae* apterae adults on pepper plants grown under aCO_2_ (400 ppm) and eCO_2_ (650 ppm).

Non-sequential variables	Treatment	PPW	NWEI	*p*	WDI	*p*	WDE	*p*	Percentage	*p*
Non-probe	aCO_2_	42/42	23.26 ± 2.30 (4–57)	**0.027**	2085.45 ± 262.35 (240.31–6967.96)	0.378	94.22 ± 5.58 (2.33–1689.09)	**0.001**		
	eCO_2_	40/40	17.03 ± 2.51 (1–64)		1936.62 ± 282.37 (139.10–7443.69)		84.24 ± 7.61 (1.30–1849.54)			
Probe	aCO_2_	42/42	23.17 ± 2.30 (4–57)	**0.028**	26699.29 ± 262.35 (21816.76–28544.55)	0.378	1174.15 ± 172.43 (9.77–27425.60)	0.369		
	eCO_2_	40/40	17.00 ± 2.51 (1–64)		26848.12 ± 282.37 (21341.07–28645.64)		1475.05 ± 211.92 (9.75–25800.66)			
pd	aCO_2_	42/42	118.60 ± 9.39 (25–256)	0.185	599.13 ± 51.79 (123.00–1560.81)	0.170	5.31 ± 0.02 (2.94–12.00)	**<0.001**		
	eCO_2_	40/40	107.05 ± 11.94 (21–301)		532.77 ± 60.20 (81.82–1615.75)		4.86 ± 0.02 (2.91–13.27)			
C	aCO_2_	42/42	27.01 ± 2.53 (4–65)	**0.017**	10041.60 ± 865.03 (2289.27–22618.31)	0.330	391.90 ± 27.53 (9.77–5813.95)	0.601	38.51 ± 3.54 (8.10–85.64)	0.344
	eCO_2_	40/40	19.35 ± 2.57 (3–66)		9109.61 ± 986.98 (1183.33–23545.48)		428.46 ± 33.30 (9.75–6750.34)		35.19 ± 4.09 (4.15–92.90)	
E1	aCO_2_	42/42	4.79 ± 0.50 (1–14)	**0.016**	1367.96 ± 198.98 (37.06–5410.55)	**0.037**	251.49 ± 30.97 (21.35–1551.16)	0.614	5.12 ± 0.74 (0.13–19.62)	**0.021**
	eCO_2_	39/40	3.35 ± 0.47 (0–14)		887.69 ± 155.08 (29.93–4178.15)		328.21 ± 61.52 (5.28–3479.72)		3.29 ± 0.62 (0.00–17.94)	
Single E1	aCO_2_	32/42	2.29 ± 0.34 (0–8)	**0.009**						
	eCO_2_	21/40	1.23 ± 0.29 (0–8)							
E2	aCO_2_	41/42	2.50 ± 0.30 (0–9)	0.385	14030.68 ± 1310.98 (33.62–25363.72)	0.272	4832.44 ± 1046.12 (9.26–25363.72)	0.386	50.22 ± 4.74 (0.00–89.82)	0.425
	eCO_2_	38/40	2.13 ± 0.25 (0–6)		15793.52 ± 1334.13 (1409.46–27163.43)		6217.17 ± 1112.24 (25.31–24282.08)		54.50 ± 4.80 (0.00–95.09)	
Probe after 1^st^ E1	aCO_2_	32/42	10.17 ± 1.83 (0–44)	**0.005**						
	eCO_2_	16/40	5.70 ± 1.62 (0–39)							
Sequential variables										
End of last pd to end of probe	aCO_2_	42/42			381.19 ± 165.28 (1.70–5788.84)	**0.005**				
	eCO_2_	40/40			1993.63 ± 942.89 (3.48–27379.65)					

*P*-values according to Mann Whitney *U*-test for non-Gaussian variables. Bold-type indicates significant differences (*p* ≤ 0.05).

PPW, proportion of individuals that produced the waveform type; NWEI, number of waveform events per insect; WDI, waveform duration (sec) per insect; WDE, waveform duration (sec) per event. Probe: probe activity. Waveforms: pd, short intracellular punctures; C, intercellular stylet pathway; E1, phloem salivation; single E1, E1 not followed by E2.
