# Supplementary material for: Elevated CO2 impacts bell pepper growth with consequences to Myzus persicae life history, feeding behaviour and virus transmission ability
Source: Sci Rep. 2016 Jan 8;6:19120. doi: 10.1038/srep19120 (PMC4705479; doi:10.1038/srep19120)
Supplement: Supplementary Information [file srep19120-s1.pdf]

SUPPLEMENTARY INFORMATION

Elevated CO_2 impacts bell pepper growth with consequences to *Myzus persicae* life history, feeding behaviour and virus transmission ability

Beatriz Dáder[1,2,*], Alberto Fereres[1], Aránzazu Moreno[1], Piotr Trębicki[2]

[1]Institute of Agricultural Sciences. Spanish National Research Council. Calle Serrano 115 dpdo, 28006 Madrid, Spain

[2]Grains Innovation Park. Department of Economic Development, Jobs, Transport and Resources. 110 Natimuk Road, Horsham VIC 3400, Australia

*beatrizdader@ica.csic.es

Organism	Parameter	aCO_2	eCO_2	*p*
Aphid	*d*	6.72±0.11	7.44±0.13	**<0.001**
	Md	39.76±1.33	28.80±1.46	**<0.001**
	M_{10}	53.52±1.42	33.76±1.89	**<0.001**
	Td	9.11±0.15	10.08±0.18	**<0.001**
	r_m	0.40±0.01	0.33±0.01	**<0.001**
	RGR	0.47±0.01	0.39±0.01	**<0.001**
Pepper	Height (cm)	12.5±0.5	14.3±0.3	**0.002**
	Number of leaves	12.9±0.6	13.4±0.3	0.418
	SPAD	43.8±0.9	28.9±1.2	**<0.001**
	Leaf area (cm^2)	487.3±37.8	421.4±22.6	0.184
	Leaf dry weight (g)	1.6±0.1	2.3±0.1	**0.001**
	Stem dry weight (g)	1.3±0.1	1.6±0.1	**0.018**
	Above-ground dry weight (g)	2.9±0.2	3.9±0.2	**0.002**
	Specific leaf area	314.3±9.8	187.8±0.1	**<0.001**
	C (%)	41.0±0.2	41.2±0.1	0.521
	N (%)	3.1±0.1	1.1±0.1	**<0.001**
	C:N	13.8±0.6	37.0±1.2	**<0.001**

Supplementary Table S1. Life history parameters of *Myzus persicae* and plant development and leaf chemical profile of peppers grown under aCO_2 (400 ppm) and eCO_2 (650 ppm). Different letters stand for statistical differences according to Student *t*-test for Gaussian variables or Mann-Whitney *U*-test for non-Gaussian variables ($p \leq 0.05$). Statistically different parameters are highlighted in bold.
